# The Feasibility of Laparoscopic Cholecystectomy in Patients with Previous Abdominal Surgery

**DOI:** 10.1155/1998/35456

**Published:** 1998

**Authors:** J. Diez, R. Delbene, A. Ferreres

**Affiliations:** 1 Department of Surgery University Hospital Av. Quintana Buenos Aires 70- 1014 Argentina; 2 Dragones Clinic Araoz Buenos Aires 191-1414 Argentina; 3 University Hospital Av. Quintana Azcuenaga Buenos Aires 1221-1115 Argentina

## Abstract

A retrospective study was carried in 1500 patients submitted to elective laparoscopic cholecystectomy to ascertain its feasibility in patients with previous abdominal surgery. In 411 patients (27.4%) previous infraumbilical intraperitoneal surgery had been performed, and 106 of them (7.06%) had 2 or more operations. Twenty five patients (1.66%) had previous supraumbilical intraperitoneal operations (colonic resection, hydatid liver cysts, gastrectomies, etc.) One of them had been operated 3 times. In this group of 25 patients the first trocar and pneumoperitoneum were performed by open laparoscopy. In 2 patients a Marlex mesh was present from previous surgery for supraumbilical hernias. Previous infraumbilical intraperitoneal surgery did not interfere with laparoscopic cholecystectomy, even in patients with several operations. There was no morbidity from Verres needle or trocars. In the 25 patients with supraumbilical intraperitoneal operations, laparoscopic cholecystectomy was completed in 22. In 3, adhesions prevented the visualization of the gallbladder and these patients were converted to an open procedure. In the 2 patients Marlex mesh prevented laparoscopic cholecystectomy because of adhesions to abdominal organs. We conclude that in  most instances previous abdominal operations are no contraindication to laparoscopic cholecystectomy.


*HPB Surgery*, 1998, Vol. 10, pp. 353–356
Reprints available directly from the publisher
Photocopying permitted by license only

© 1998 OPA (Overseas Publishers Association)
Amsterdam B.V. Published under license
under the Harwood Academic Publishers imprint,
part of The Gordon and Breach Publishing Group.
Printed in India.

### The Feasibility of Laparoscopic Cholecystectomy in Patients with Previous Abdominal Surgery

J. DIEZ[a,*], R. DELBENE[b,†] and A. FERRERES[a,‡]

[a]*Department of Surgery, University Hospital, B. A. Argentina;* [b]*Dragones Clinic*

*(Received 28 October 1996)*

**A retrospective study was carried in 1500 patients submitted to elective laparoscopic cholecystectomy to ascertain its feasibility in patients with previous abdominal surgery. In 411 patients (27.4%) previous infraumbilical intraperitoneal surgery had been performed, and 106 of them (7.06%) had 2 or more operations. Twenty five patients (1.66%) had previous supraumbilical intraperitoneal operations (colonic resection, hydatid liver cysts, gastrectomies, etc.) One of them had been operated 3 times. In this group of 25 patients the first trocar and pneumoperitoneum were performed by open laparoscopy. In 2 patients a Marlex mesh was present from previous surgery for supraumbilical hernias. Previous infraumbilical intraperitoneal surgery did not interfere with laparoscopic cholecystectomy, even in patients with several operations. There was no morbidity from Verres needle or trocars. In the 25 patients with supraumbilical intraperitoneal operations, laparoscopic cholecystectomy was completed in 22. In 3, adhesions prevented the visualization of the gallbladder and these patients were converted to an open procedure. In the 2 patients Marlex mesh prevented laparoscopic cholecystectomy because of adhesions to abdominal organs. We conclude that in most instances previous abdominal operations are no contraindication to laparoscopic cholecystectomy.**

*Keywords:* Laparoscopic cholecystectomy, previous surgery

### INTRODUCTION

Previous abdominal surgery may increase the difficulty of laparoscopic cholecystectomy [20, 16, 17] especially in upper abdominal operations [11, 15].

For this reason, it has been considered to be a relative contraindication in the past [1, 13, 14, 18].

With experience, many surgeons, have found that the procedure can often be safely performed, but there have been few studies that have examined this subject in detail [2, 5, 7, 16, 19].

The purpose of this retrospective study is to examine the influence of previous upper and lower abdominal surgery on the performance of laparoscopic cholecystectomy in a University and private clinic.

### MATERIAL

The records of 1500 consecutive patients treated by laparoscopic cholecystectomy between De-

*Av. Quintana 70 - 1014 - Buenos Aires, Argentina Fax.: 54-1-812-1140.
†Araoz 191 - 1414 - Buenos Aires, Argentina.
‡Azcuenaga 1221 - 1115 - Buenos Aires, Argentina.

cember 1990 and October 1995 were reviewed. The mean age was 43 ranging form 7 to 88: There were 1215 females and 385 males; 52 patients were over 70 years of age.

Previous infraumbilical intraperitoneal surgery was recorded in 411 patients (27.4%). In 106 of them, 2 or more operations had been performed. Cesarean was the most common procedure followed by appendectomy and gynecological operations. In 25 patients (1, 66%) previous supraumbilical intraperitoneal operations had been performed.

(Tab. I) Three of them had had 2 previous procedures; while another had a history of splenectomy, reexploration for hemoperitoneum and another laparotomy to recover a foreign body (sponge) in the splenic area.

In 2 patients a Marlex mesh had been used to treat an epigastric hernia.

In patients with infraumbilical scars, pneumoperitoneum was created using a Verress needle. The first trocar was inserted just above the umbilicus by a blind technique. In patients with prior supraumbilical operations an open laparoscopy was performed.

TABLE I Previous supraumbilical intraoperative intraperitoneal surgery (n 25)

| | |
|---|---|
| Gastrectomy | 2 |
| Vagotomy and pylorplasty | 1 |
| Left colectomies | 2 |
| Splenectomy | 2 |
| Hydatid liver disease | 1 |
| Small bowell resection | 2 |
| Diaphragmatic hernia repair | 1 |
| Abdominal trauma: left transverse colectomy+transverse colostomy | 1 |
| Retained foreign body | 1 |
| Supraumbilical laparotomy | 1 |
| Hemoperitoneum | 1 |
| Generalized peritonitis | 4 |
| Pancreatitis | 3 |
| | 22 |
| **Converted** | |
| Splenectomy + Reoperation Hemoperitoneum + Reoperation Foreign body (Sponge) | 1 |
| Pancreatitis + Reoperation Hemoperitoneum | 1 |
| Gastrectomies + Reoperation Peritonitis | 1 |
| | 3 |

In both cases other trocar sites were chosen under visual control. Once inside the peritoneal cavity, adhesions were divided under direct vision and laparoscopic cholecystectomy was performed.

### RESULTS

The overall rate of conversion to open cholecystectomy in 1500 patients was 2.26%. Indications for conversion were due to the original pathology in 27 patients (18%) and in 7(0.4%) to intraoperative accidents or problems. (Tab. II)

No intraoperative or postoperative complications resulted from the insertion of the Verress needle or the trocars.

In patients with infraabdominal operations even with more than one prior operation, there was no increased difficulty in performing laparoscopic cholecystectomy. In 3 of the 25 patients with previous upperabdominal surgery adhesions prevented visualization of the biliary and vascular elements and open surgery was performed (8, 6%) after conversion. These 3 patients had several operations for generalized peritonitis and hemoperitoneum.

We attempted to perform laparoscopic cholecystectomy in 2 patients with Marlex meshs in the upper abdomen used to treat an epiagtric hernia. We failed; trocars could not enter the mesh and extensive adhesions prevented visua-

TABLE II Conversion to open surgery

| | | |
|---|---|---|
| Inadequate visualization of structures | 14 | (1, 8%) |
| Adhesions | 6 | |
| Mirizzi syndrome | 4 | |
| Cholecystoduodenal fistula | 1 | |
| Choledocolithiasis | 2 | |
| Cyctic artery injury | 1 | (0, 40%) |
| Liver bed hemorrhage | 2 | |
| Bile duct injury | 1 | |
| Epigastric artery injury | 1 | |
| Morbid Obesity | 1 | |
| Equipment failure | 1 | |
| | 34 | |

lization of the biliary structures. Both patients were converted and open cholecystectomy performed.

### DISCUSSION

Previous abdominal surgery was once considered a contraindication to laparoscopic cholecystectomy. It was thought that adhesions of organs to the abdominal wall might increase the chance of puncture or laceration by trocars [14-18]. Patients with upper abdominal incisions were considered at higest risk of this complication [17]. In was this group in whom the laparoscopic technique was considered unsafe and therefore excluded [8, 11, 15, 20].

Abdominal surgeons failed to observe that gynecologists routinely performed several laparoscopic procedures on their patients with almost no inconvenience due to previous operations. Semm published statistics of the Federal Republic of Germany of Laparoscopic Gynecological from 1983-5 and found that complications were generally due to lack of experience of the surgeons and not to previous surgery [14]. With more experience, previous abdominal surgery, even supramesocolonic was considered a relative contraindication. In patiens with previous operations in the lower abdomen Olsen [10] and Semm [14] recomend, the introduction of the laparoscope, trough a trocar at the midclavicular site and then placement of the umbilical port under direct vision.

In patients with upper abdominal surgery an open insertion of the first trocar is recomended [3-10]. Pellegrini [12] Yu [20] and Wongworrabat [19] have stated that previous abdominal surgery is no longer a contraindication for laparoscopic cholecystectomy N.I.H. Consensus Statement:" Gallstones and Laparoscopic Cholecystectomy" published in 1992, does not include previous surgery as a contraindication [9]. Caprini recommends intraoperative ultrasound to detect intraabdominal adhesions.

It is now accepted that in patients with preivous abdominal surgeries, laparoscopy should be tried although it will have a higher conversion rate [4-6].

### CONCLUSIONS

Although authors have now recommended that the laparoscopic technique be attempted in patients with previous surgery there, have been few series documenting results in such patients. Our study confirms that the producere is safe. It will result in few conversions if the prior surgery has been in the lower abdomen. With upper abdominal operations, conversion will be more frequent, but not less safe.

When Marlex mesh has been inserted conversion rates will likely be quite high because of the extensive adhesions associated with this material when it is left in contact with abdominal viscera.

### *References*

[1] Bonatsos, G., Leandros, E. and Dourakis, N. *et al.* (1995). "Laparoscopic cholecystectomy. Intraoperative finding and postoperative complications". *Surg. Endosc.*, **9**, 889-93.

[2] Caprini, J., Areelus, J. and Swanson, J. *et al.* (1995). "The ultrasound localization of abdominal wall adhesions" *Surg. Endosc.*, **9**, 283-5.

[3] Cuschiery, A. and Berci, G. (1990). "Laparoscopic Biliary Surgery". Blackwell Scientific Publications, Oxford, 43.

[4] Decoud, J., Kaplan, J. and Merganta, P. *et al.* (1991). "Colecistectomía laparoscópia" *Rev. Arg. Circ.*, **61**, 45-62.

[5] Freys, S., Frechs, H. and Heimbucher, J. (1994). "Laparoscopische Eingriffe ber voroperierten Patienten" *Chirurg.*, **65**, 616-23.

[6] Klee, F., Oswald, B. and Wysocki St. (1995). "Factors influencing the decision to convert from laparoscopic to conventional cholecystectomy". European I.H.P.B.A. Congress Athens" 95, 335/339, Monduzzi Editore S.P.A. Bologna Italy.

[7] Liu, Cl., Fan, S. and Lai, E. *et al.* (1996). "Factors influencing conversion of laparoscopic cholecystectomy to open surgery." *Arch. Surg.*, **131**, 98-101.

[8] Moosa, R. (1993). "Tratamiento quirúrgico de la litiasis en el año 1992" *Arch. Arg. Enf. Ap. Dig.*, **7**(1), 94-9.

[9] N.I.H. Consensus Statement: "Gallstones and laparoscopic cholecystectomy" 1992, **10**, 3.

[10] Olsen, D. (1991). "Laparoscopic Cholecystectomy" *The Am. Jour of Surg.*, **161**, 339-44.

[11] Perissat, J., Collet, D. and Belliard, R. *et al.* (1992). "Laparoscopic Cholecystectomy: The state of the art. A report of 700 cases" *World j. Surg.*, **16**, 1074–82.
[12] Pellegrini, C. (1994). "Cirugía Videoscópica" Relato Oficial 65° Congreso Argentino de Cirugía. *Rev. Arg. Cir.* 33–41. Número extraordinario.
[13] Res, A., Sarr, M. and Nagorney, D. (1993). "Spectrum and management of major complications of laparoscopic cholecystectomy". *Am. J. Surg.*, **156**, 655.
[14] Riedel, H., Lehmann-Willembrock, E., Meche, H. y Sem, K. (1989). The frequency distribution of pelvisicopc (laparoscopic) operations, including complications rates: Statistics of Federal Republic of Germany in the years 1983–5". *Zent. Cl. Gynakol.*, **2**, 78.
[15] Roseau, E. (1989). La cholecystectomie par coelioscopie, une tecnique d'exception" *La Presse Medic.*, **18**, 1528.
[16] Schirmer, B., Dix, J. and Schiez, R. *et al.* (1995). The impact of previous abdominal surgery on out come following laparoscopic cholecystectomy" *Surg. Endosc.*, **9**, 1085–9.
[17] Soper, N. (1991). "Laparoscopic Cholecystectomy: A promising new branch in the algorithm of gallstone managent" *Surgery*, **109**(3), 342–4.
[18] Terblanche, J. (1991). "Laparoscopic cholecystectomy: A new milestone or a dangerous innovation?" *H.P.B. Surgery*, **3**, 177–80.
[19] Wongworawat, M., Aitken, D. and Robles, A. (1994). "The impact of prior abdominal surgery on laparoscopic cholecystectomy" *Am. Surg.*, **60**, 763–6.
[20] Yu, S., Chen, S. and Wang, S. (1994). "Is previous abdominal surgery a contraindication to laparoscopic cholecystectomy?" *J. Laparoscoendosc. Surg.*, **4**, 31–5.

